# Supplementary material for: The Dual Prey-Inactivation Strategy of Spiders—In-Depth Venomic Analysis of Cupiennius salei
Source: Toxins (Basel). 2019 Mar 19;11(3):167. doi: 10.3390/toxins11030167 (PMC6468893; doi:10.3390/toxins11030167)
Supplement: Supplementary file 1 [file toxins-11-00167-s001.zip › Supplementary Fig S1.1-S1.19/Supplementary Fig S1.10.pdf]

Supplementary Fig S1.10

CLUSTAL O(1.2.4) multiple sequence alignment: CRISP1s & CRISP2s

```
tr_E9IAI6_SOLIN             MELIV------------SIL---WLAI--TAE----NLANTLATNYCNLQSCKRNNAIHT
sp_Q05110_VESVU             MEI-S------------GLV---YLIIIVTII----DLPYGKANNYCKIKCL--KGGVHT
sp_P35759_POLEX             MEI-G------------GLV---YLVIVVAII----HSSQ--GVDYCKIRCP--S-GIHT
tr_A0A131YIC3_RHIAP         MTVLRLQLARYLFLLLPSLL----LVLVLPAVP--KGEAAPIVTSNCSETF-LRYNQNHT
tr_L7MBC4_RHIPU             MKLNR---GKPAHLRVTEMALAVFFTLSAPVAVARV--VDNSGQSVCEDIY-RRYNENHV
tr_G3MIH5_AMBMA             ----------------QVRAPPSLASLITRAVDGRITTGGSQNNGECESIY-LRYNQDHV
tr_A0A023GBM8_AMBTR         MQLKQ---VRPKTCLARCVHLLVLLSLITRAVDGRITTGGSQNNGECESIY-LRYNQDHV
CRISP1_a1_CUPSA             ----M--SAP---ARIR------NILLVLSSV---LCTLTVIQSAYCPDIY-KRYSTQHS
CRISP2_CUPSA                -------MGI---PSLF------FLVTILVSY---TT---AQTTPGCPALF-LRYSKTHS
tr_T1DPC1_LYCBU             MGGIS--SGD---VRLH------ALALLLLAV---GW---AGAVSHCPALY-LRYSNRHT
tr_T1DEK7_ISOVE             MGGIS--SGD---ARLH------ALALLLLTV---GW---AGAVSHCPALY-LRYSNRHT
tr_A0A1E1WVV1_TITOB         MKEL---------LLLF------LKLVVLFLY---LC---SSVLSECPALY-RRYSKEHT
tr_A0A0C9RP88_TITBA         MKGI---------LQLF------LKLVVLFVY---LC---SSVLSECPDLY-RRYSKEHT
                                                                          *           *

tr_E9IAI6_SOLIN             MCQYTSPT-PGPMCL--EYSNVGFTDAEKDAIVNKHNELRQRVASGKEMRGTNGPQPPAV
sp_Q05110_VESVU             ACKYGSL---KPNCGNKVVVSYGLTKQEKQDILKEHNDFRQKIARGLETRGNPGPQPPAK
sp_P35759_POLEX             VCQYGESTKPSKNCAGKVIKSVGPTEEEKKLIVSEHNRFRQKVAQGLETRGNPGPQPAAS
tr_A0A131YIC3_RHIAP         MCREE-P-----AC---EIQVAGIDDNVKQLILKLHNHYRSLIAGGNET-----HMPPAA
tr_L7MBC4_RHIPU             LCRLPES-----AC---QPVASGVGDADKDIILAAHNHYRSQIAIGNNS-----RFPHAS
tr_G3MIH5_AMBMA             MCRQHAS-----TC---QPIASGIEDSDKDKILIAHNRYRSQIATGNTT-----RLPPAA
tr_A0A023GBM8_AMBTR         MCRQHAS-----TC---QPIASGIEDSDKDKILIAHNRYRSQIATGNTT-----RLPPAA
CRISP1_a1_CUPSA             YCLGQCC-----NC---TFYKRGVDPGLKGQILSLHNDLRNKVAAGHSYGVD--HLPRAS
CRISP2_CUPSA                YCKPANP-----AC---NGMRSSVSAQDMNAILKEHNTHRSKLATGGEQ-----NLPTAA
tr_T1DPC1_LYCBU             FCQSKND-----KC---QIKRSGVTLDERDTILELHNSIRNEIALGKDASGK--RLPHAG
tr_T1DEK7_ISOVE             FCQPKNE-----KC---QIKRSGVTLDDRDTILELHNGIRNEIALGKDESGK--NLPHAG
tr_A0A1E1WVV1_TITOB         FCKTKNQ-----KC---HIKRWGVSEDDREIIIDLHNKVRNNIALGKDQSG---RLPAAG
tr_A0A0C9RP88_TITBA         FCKTKNQ-----KC---NIKRWGVSQDDRDIIINLHNRVRNNIALGQDQSG---RLPAAG
                             *           *        .        *:  **  *. :* *          * *

tr_E9IAI6_SOLIN             KMPNLTWDPELATIAQRWANQCTF-EH--DACRNVERF-AVGQNIAATSSS---GKNKST
sp_Q05110_VESVU             NMKNLVWNDELAYVAQVWANQCQY-GH--DTCRDVAKY-QVGQNVALTGST---AAKYDD
sp_P35759_POLEX             DMNDLVWNDELAHIAQVWASQCQFLVH--DKCRNTAKY-PVGQNIAYAGGS-----KLPD
tr_A0A131YIC3_RHIAP         NMLEMEWDDDLARVAQAHANLCQF-EHDCPSCRSLQKFTDVGQNLCLDRTT--RDDPQPD
tr_L7MBC4_RHIPU             NMLEMEWDDDLARVAQAHANLCSG-QPSCSSCIRIEKFPHVGRTGCLIRTD--SENNTAD
tr_G3MIH5_AMBMA             NMMELEWDDDLARVAQAHANLCAN-QPSCSSCMRIEKFPIVGRTGCLIRTD--NEDNNRD
tr_A0A023GBM8_AMBTR         NMMELEWDDDLARVAQAHANLCAN-QPSCSSCMRIEKFPIVGRTGCLIRTD--NEDNNRD
CRISP1_a1_CUPSA             NMLEMVWDDELATIAQTWLDGCRK-NGDCEECRMVSRF-AVGQEIIGDFIGSEMD--SQD
CRISP2_CUPSA                NMLEMVWDDELAAVAQKHADQCLF-EHDCSTCRRVQNY-GVGQNLAIEQVSNSPSIPSPN
tr_T1DPC1_LYCBU             NMLEMEWDEELAQVAQKLADRCIF-NHDCDECRKVENF-DVGQNLYTATVT-ATKPPHPS
tr_T1DEK7_ISOVE             DMLEMEWDEELAQVAQKLADRCVF-NHDCNECRKVENF-DVGQNLYMATVI-ATKPPHPS
tr_A0A1E1WVV1_TITOB         DMLEMEWDDELAQIAQKLTDQCVY-KHDCDDCRKVENF-DVGQNIYTATIT-AVDPPEPF
tr_A0A0C9RP88_TITBA         DMLEMEWDEELAQIAQKLADQCVF-KHDCDECRKVENF-DVGQNIFTTTIT-AVEIPDPF
                            .* :: *: :** :**   . *         *    .:  **:

tr_E9IAI6_SOLIN             PNEMILLWYNEVKDFDN-RWISSFPSDDNILMKVGHYTQIVWAKTTKIGCGRIMFKEPDN
sp_Q05110_VESVU             PVKLVKMWEDEVKDYNP-KKK----FSGNDFLKTGHYTQMVWANTKEVGCGSIKYIQ-EK
sp_P35759_POLEX             VVSLIKLWENEVKDFNY-NTG----ITKQNFAKIGHYTQMVWGKTKEIGCGSLKYME-NK
tr_A0A131YIC3_RHIAP         WESCVRRWYDEVSLFSN-ASRTPFQF----DLPTGHFTQMVWATTWKIGCGYTRYPSTEP
tr_L7MBC4_RHIPU             WEGCIGYMYKESLRIPNTSSSTPLRT----MRGIESFTQLAWATTWKVGCGYAKYPSG-S
tr_G3MIH5_AMBMA             WEGCIGHMFEEHSRLPDTGLNTPIRT----ARGAESFTQLAWATTWRVGCGYVKYPSQRS
tr_A0A023GBM8_AMBTR         WEGCIGHMFEEHSRLPDTGLNTPIRT----ARGAESFTQLAWATTWRVGCGYVKYPSQRS
CRISP1_a1_CUPSA             LDNEFFSRFNALSVFTD-AHVREYSD----SNGNHKTSQLLWAKTWRIGCGFLRFLDPNN
CRISP2_CUPSA                WPSAIKSWYDEIAYFSS-NAIDPFIA-PPPEPTYGHFSQLLWATSWRIGCGYVLYQDR-N
tr_T1DPC1_LYCBU             WKDAIQAWYSEVHRFSN-DLINPFTS----DDATGHFTQMVWSNTWKIGCGFVSYEKRKD
tr_T1DEK7_ISOVE             WKDAIKAWYSEVHRFSN-DLINPFTS----DGATGHFTQMAWANTWKIGCGFVSYEKRKD
tr_A0A1E1WVV1_TITOB         WVDAVRSWYSEIYRFTP-DFIKPFTS----DHATGHFTQMAWSKTWRVGCGYVLYEKRKD
tr_A0A0C9RP88_TITBA         WVDAIRSWYSEIYRFTP-DFIKPFTS----DHATGHFTQMVWSKTWRVGCGYVLYEKRKD
                                .    .                           :*: *..: .:***   : .

tr_E9IAI6_SOLIN             W--TKHYLVCNYGPAGNVLGAPIYEIKK--------------------------------
sp_Q05110_VESVU             W--HKHYLVCNYGPSGNFMNEELYQTK---------------------------------
sp_P35759_POLEX             M--QNHYLICNYGPAGNYLGQLPNTKK---------------------------------
tr_A0A131YIC3_RHIAP         PFTYDLLYTCDYGPGGNIVGGEMYEEGDACSNCPEGTCCGSSCEEQNVDTRFKGLCKPTT
tr_L7MBC4_RHIPU             ALRFEKLYTCAYGPGGNVRGEQVYKVGPACSDCPEGTCCGVSCRRYGFTPAHSGLCTVAG
tr_G3MIH5_AMBMA             GLRYEKLYTCAYGPGGNTRGEDVYKVGQPCSDCPEGTCCGSSCRRYGILQSYEGLCKTTG
tr_A0A023GBM8_AMBTR         GLRYEKLYTCAYGPGGNTRGEDVYKVGQPCSDCPEGTCCGSSCRRYGILQSYEGLCKTTG
CRISP1_a1_CUPSA             GRTVVNILLCNYGPGGCVEGEEVYKAGTVCSACPANTCCGDGCKKHKITNSFKGLCKVID
CRISP2_CUPSA                M--YQKLYVCNYGPAGNTVSHSMYKVGPPCSACPINSCCDRTCGAQK---EYSGLCKITD
tr_T1DPC1_LYCBU             S--WTQLYVCNYGPGGNVDDDEMYMIAQACSKCPGNTCCGMHCKKRKS-PSYVGLCKVLN
tr_T1DEK7_ISOVE             S--WNQLYVCNYGPGGNVDDNEMYKIAHACSKCPGNTCCGMHCKKRKS-ASYVGLCKVLN
tr_A0A1E1WVV1_TITOB         S--WTQLYVCNYGPAGNMDDSELYKIDKPCEKCPGNTCCGPHCKKKRS-TSYLGLCSVLN
tr_A0A0C9RP88_TITBA         S--WTQLYVCNYGPAGNMDDSELYKVDQPCEKCPSNTCCGSHCKKKRT-TSYLGLCDVLN
                                     * ***.*   .

tr_E9IAI6_SOLIN             ------------------------------------------------------------
sp_Q05110_VESVU             ------------------------------------------------------------
sp_P35759_POLEX             ------------------------------------------------------------
tr_A0A131YIC3_RHIAP         PEGPKPS---VS-KKGLIWACLFNNETAESCETVSDPPNAFNTKPLFGAGYLETVVEGGN
tr_L7MBC4_RHIPU             -DGPGVL---LDDEDNLVFSCLFNRVTSRWCQFRSDPNDGWTTKTFFATGYAESVLQAGQ
tr_G3MIH5_AMBMA             -DGPSPT---LD-EDDLVLSCLFNRATSRFCRYRSEPADGFTTKNFFARGVVESVLQSGQ
tr_A0A023GBM8_AMBTR         -DGPSPT---LD-EDDLVLSCLFNRATSRFCRYRSEPADGFTTXXFFARGIVESVLQSGQ
CRISP1_a1_CUPSA             ENLPPQGNVPHVKSGKELFYCGFNG--ESDCSRAVEGINRWIGKISTGGTWLSMDLGRGG
CRISP2_CUPSA                NKAPNYK---P--VGSYMFYCDFNN--QSDCNGYIIGTNKWLTYNTLSGSFLGIVLNGGE
tr_T1DPC1_LYCBU             EKGPDYD---EGDFSHFVFNCDFKTESTSDCQIHVDGTNKWQTRQVVSGVYKTVVLNGGE
tr_T1DEK7_ISOVE             KKGPDYD---EGDFSHFVFNCDFKTESASDCQIQVDGTNKWQTRQVVSGVYKTVVLNGGE
tr_A0A1E1WVV1_TITOB         GRGPDFD---DIDFSNYIFNCDFRPESSSDCNNKVEGSNKWQTRQIISDVYKTVVLNGGE
tr_A0A0C9RP88_TITBA         GSGPDFD---ETDFSNYIFNCDFRPESSSDCNNKVEGSNKWQTRQIISDVYKTIVLNGGE

tr_E9IAI6_SOLIN             ------------------------------------------------------------
sp_Q05110_VESVU             ------------------------------------------------------------
sp_P35759_POLEX             ------------------------------------------------------------
tr_A0A131YIC3_RHIAP         RVELTFKRTIKS-PQSPFCINIEYAKGPSMAGDKADGSLRLLLTSPVLNL-----FQIDA
```

```
tr_L7MBC4_RHIPU          SAEMTFERPIQPSSNGLLCVEVDYSNGPNVAGTPDRGMLELVVTPVDRPE-----RQRTI
tr_G3MIH5_AMBMA          SAEVTLEQPVRS-SSGPLCVEVEYSKGPNVAGQPDRGLFNLIVTPVNRPE-----RQRTI
tr_A0A023GBM8_AMBTR      SAEVTLEQPVRS-SSGPLCVEVEYSKGPNVAGQPDRGLFNLIVTPVNRPE-----RQRTI
CRISP1_a1_CUPSA          HTSLTFEKPIKS-KSGKLCLSITTRTGPVDANSRYTYELGGSMEETGRFLTTMVFPKADN
CRISP2_CUPSA             ESTMVFNNKIKP-STSAFCFVVMYRKGPNVDGQEESNSATETFSGSNFEFQQNLPTFIGD
tr_T1DPC1_LYCBU          STTLKFTSHIQ--SKDGFCLTIAFRKGPNVAGAKNANEFSVQLERKGA--TKPLNFQLDS
tr_T1DEK7_ISOVE          STTLKFTSHIQ--SKDGFCLTLAFRKGPNVAGTKNDNEFNVLLERKGA--TKPLNFRLDS
tr_A0A1E1WVV1_TITOB      STALKFTSNIQ--YKDGFCLTISFRKGPSVAGTKSAGEFNVQLERKGA---SPLNFQLDS
tr_A0A0C9RP88_TITBA      NTVLKFTSNIQ--SKDGFCLTISFRKGPNIAGTNNVGEFDVQLERKGA---KPLNFRLDS

tr_E9IAI6_SOLIN          ------------------------------------------------------------
sp_Q05110_VESVU          ------------------------------------------------------------
sp_P35759_POLEX          ------------------------------------------------------------
tr_A0A131YIC3_RHIAP      ELGGDYTGYQMYSFSLDFRIPVQVGFSYSVPANSSAQYFSLYKLIVNQGACSQNF-----
tr_L7MBC4_RHIPU          SLSEGATNMMRMRITLNYSVRVKIGFSFMVPRGSPAQYANIHKVAIYERACSE-------
tr_G3MIH5_AMBMA          NLSGSATNIVIMRVTLNYDVPVQIGFSFVVPGGSQAQYVNIHKIAIYEKSCSV-------
tr_A0A023GBM8_AMBTR      NLSGSSTNIVIMRVTLNYDVPVQIGFSFVVPGGSQAQYVNIHKIAIYEKSCSV-------
CRISP1_a1_CUPSA          KVKHRFHTDYFQPSEFPKNRDVKLTLSFSVPIDGQRQYIEIKRIVAMEKDCSKQTILEGS
CRISP2_CUPSA             -TRHQF---SQFSMSLAWNMETEIKITFAVPVGKPEQFLEIQSIYATEGSCKQ-------
tr_T1DPC1_LYCBU          -EGSDW---LPYSIGIPMKQPMQISLKFGVPKGAPTQYLDVKYVRARPGLCS--------
tr_T1DEK7_ISOVE          -EGSDW---LPYSIGIPMKQPIQISLKFSVPKGAPTQYLDVKYVRARPGLCS--------
tr_A0A1E1WVV1_TITOB      -EGNEW---LPYSMGIPMKQPMLINLKFSVPKGSPAQYLDISYIRARPGVCN--------
tr_A0A0C9RP88_TITBA      -EGNQF---LPYNMGIPMNHPMQINIKFNVPKGAPAQYLDISYIRARPGLCK--------

tr_E9IAI6_SOLIN          -----
sp_Q05110_VESVU          -----
sp_P35759_POLEX          -----
tr_A0A131YIC3_RHIAP      -----
tr_L7MBC4_RHIPU          -----
tr_G3MIH5_AMBMA          -----
tr_A0A023GBM8_AMBTR      -----
CRISP1_a1_CUPSA          GYSVD
CRISP2_CUPSA             -----
tr_T1DPC1_LYCBU          -----
tr_T1DEK7_ISOVE          -----
tr_A0A1E1WVV1_TITOB      -----
tr_A0A0C9RP88_TITBA      -----
```

Percent Identity Matrix - created by Clustal2.1

1:tr_E9IAI6_SOLIN	100.00	44.39	45.91	36.41	27.31	26.17	26.94	30.52	32.38	37.96	36.57	32.71	35.05
2:sp_Q05110_VESVU	44.39	100.00	57.21	37.62	26.79	28.37	28.77	26.57	33.33	33.97	32.54	32.85	32.85
3:sp_P35759_POLEX	45.91	57.21	100.00	34.15	25.49	24.63	25.12	29.56	33.17	33.33	33.33	33.66	34.65
4:tr_A0A131YIC3_RHIAP	36.41	37.62	34.15	100.00	40.75	42.21	41.63	29.19	35.48	38.05	37.80	36.21	34.73
5:tr_L7MBC4_RHIPU	27.31	26.79	25.49	40.75	100.00	66.82	65.29	27.78	30.10	33.01	33.25	31.03	31.77
6:tr_G3MIH5_AMBMA	26.17	28.37	24.63	42.21	66.82	100.00	97.18	28.54	31.66	30.50	30.50	29.75	30.75
7:tr_A0A023GBM8_AMBTR	26.94	28.77	25.12	41.63	65.29	97.18	100.00	28.12	31.27	30.24	30.24	29.73	30.47
8:CRISP1_a1_CUPSA	30.52	26.57	29.56	29.19	27.78	28.54	28.12	100.00	30.73	32.37	32.61	32.35	32.35
9:CRISP2_CUPSA	32.38	33.33	33.17	35.48	30.10	31.66	31.27	30.73	100.00	40.29	41.52	39.45	41.94
10:tr_T1DPC1_LYCBU	37.96	33.97	33.33	38.05	33.01	30.50	30.24	32.37	40.29	100.00	92.18	68.75	68.51
11:tr_T1DEK7_ISOVE	36.57	32.54	33.33	37.80	33.25	30.50	30.24	32.61	41.52	92.18	100.00	68.03	67.31
12:tr_A0A1E1WVV1_TITOB	32.71	32.85	33.66	36.21	31.03	29.75	29.73	32.35	39.45	68.75	68.03	100.00	87.02
13:tr_A0A0C9RP88_TITBA	35.05	32.85	34.65	34.73	31.77	30.75	30.47	32.35	41.94	68.51	67.31	87.02	100.00

Orange background: insects

1:tr_E9IAI6_SOLIN: *Solenopsis invicta* (Red imported fire ant)

2:sp_Q05110_VESVU: *Vespula vulgaris* (Yellow jacket, wasp)

3:sp_P35759_POLEX: *Polistes exclamans* (Paper wasp)

Gray background: ticks

4:tr_A0A131YIC3_RHIAP *Rhipicephalus appendiculatus* (Brown ear tick)

5:tr_L7MBC4_RHIPU *Rhipicephalus pulchellus*

6:tr_G3MIH5_AMBMA *Amblyomma maculatum* (Gulf Coast tick)

7:tr_A0A023GBM8_AMBTR *Amblyomma triste*

White background: spider

8:CRISP1_a1_CUPSA [MH766629]: *Cupiennius salei*

9:CRISP2_CUPSA [MH766631]: *Cupiennius salei*

Green background: scorpions

10:tr_T1DPC1_LYCBU *Lychas buchari*

11:tr_T1DEK7_ISOVE *Isometroides vescus*

12:tr_A0A1E1WVV1_TITOB *Tityus obscurus* (Amazonian scorpion) *(Tityus cambridgei)*

13:tr_A0A0C9RP88_TITBA *Tityus bahiensis* (Brazilian scorpion)

Supplementary Fig S1.10 Multiple sequence alignment of CRISPs from insects, ticks, *C. salei*, and scorpions. Signal peptides are marked in black characters, cysteines in white with a black box, positively charged amino acids in pink, negatively charged amino acids in blue, hydrophobic amino acids in red and polar amino acids in green.
